# 

**Published:** 1888-05-12

**Authors:** 


### CONTENTS.

**Popular Fallacies about Cancer** - - - - - 85

**Words of Consolation.**—LXXVI. - - - - - 86

**Labourers' Wages and Labourers' Homes.** By the Right Hon. Earl Grey, K.G., G.C.M.G. - - - 87

**Round about the Asylums.**—VI. - - - - - 88

**Aids to Nursing.** By M. M. Basil, M.A., M.B., Author of "The Commoner Accidents to Life and Limb" - - - 89

**The Daneries:** The British Home for Incurables - - 91

**Watchman, What of the Night?** - - - - 91

**Notes and News** - - - - - - - 92

**Everybody's Page** - - - - - - - 94

**Annotations.**—Sources of Small-pox.—Calmer Weather at the Rotherham Hosp tal.—A Real University for London - - 95

**Patients' Contributions to Hospitals.** By Mr. W. Burdett-Coutts, M.P. - - - - - - - 96

**An Ishmaelite.** By Leslie Keith, Author of "The Chilcotes," "East and West," etc. - - - - 98

**The Editor's Letter-box** - - - - - 99

**Amusements with Profit for all Ages.**—For Men and Women.—Children's Corner.—Puzzles, etc. - - - 100

**EXTRA SUPPLEMENT.—THE NURSING MIRROR.**

En Passant.—Homes in the Hills.—Medical Women.—Lewes Infirmary.—Vaccination.—The Women's International Council.—Convalescent Children —Blackmailing by Nurses.—A Word of Advice.—Probationers.—Early Efforts - - - - xvii
The National Pension Fund for Nurses.—What the Nurses are Saying.—An Example to be Followed.—A Word to the Older Nurses.—Sober Sense.—Honest Difficulties - - - xviii
Appointments.—Well deserved Chastisement—Exchange and Mart xix
Every body's Opinion.—Vacancies. –Anecdotes of Personal Adventure.—Notes and Queries - - - - - - - xx

